# Cloud Detection Method Based on All-Sky Polarization Imaging

**DOI:** 10.3390/s22166162

**Published:** 2022-08-17

**Authors:** Wunan Li, Yu Cao, Wenjing Zhang, Yu Ning, Xiaojun Xu

**Affiliations:** 1 College of Advanced Interdisciplinary Studies, National University of Defense Technology, Changsha 410073, China; 2 School of Mathematics and Physics, Qingdao University of Science & Technology, Qingdao 266061, China; 3 State Key Laboratory of Pulsed Power Laser Technology, National University of Defense Technology, Changsha 410073, China; 4 Hunan Provincial Key Laboratory of High Energy Laser Technology, National University of Defense Technology, Changsha 410073, China

**Keywords:** all-sky polarization imaging, cloud assessment, polarization degree, atmospheric scattering, image fusion

## Abstract

Sky cloud detection has a significant application value in the meteorological field. The existing cloud detection methods mainly rely on the color difference between the sky background and the cloud layer in the sky image and are not reliable due to the variable and irregular characteristics of the cloud layer and different weather conditions. This paper proposes a cloud detection method based on all-sky polarization imaging. The core of the algorithm is the “normalized polarization degree difference index” (*NPDDI*). Instead of relying on the color difference information, this index identifies the difference between degree of polarization (*DoPs*) of the cloud sky and the clear sky radiation to achieve cloud recognition. The method is not only fast and straightforward in the algorithm, but also can detect the optical thickness of the cloud layer in a qualitative sense. The experimental results show a good cloud detection performance.

## 1. Introduction

Clouds play an essential role in the Earth’s energy conservation and are a critical factor in global climate change. The generation and evolution of clouds lead to sunshine and changes in temperature and relative humidity, which strongly affect the balance of the Earth’s climate system [1,2,3]. Therefore, accurate cloud cover detection has always been a crucial element of meteorological observation and is of considerable significance to many fields influencing the national economy, such as flight security, weather forecasting, and climate research. Since the traditional cloud detection methods mainly rely on professional observers, they suffer from strong subjectivity and poor accuracy. Therefore, there is an urgent need for an automatic cloud detection method that is more precise and stable, but so far, there is no reasonable solution to satisfy the users.

All-sky imagers (ASIs) working in the visible wavelength are increasingly being used across the world to detect and evaluate clouds [4,5,6,7]. However, the existing ASI equipment has two major drawbacks: (1) sun-blocking devices are used to avoid the information loss caused by overexposure in sunny areas, which reduces the accuracy and completeness of the detection results of the all-sky clouds, and (2) with the sun tracker/positioner integrated, the cost and complexity of the ASI increase greatly, preventing its widespread use [8,9]. Tao fa et al. [10] proposed a new all-sky camera (ASC) that does not cast shadow. The fisheye lens is used to capture all-sky clouds, and a light cut module is used as the sun-blocking device. The core component of the light cut module is a black chopper that can be automatically turned on and off according to the solar radiation intensity. All-sky cloud information due to overexposure is essentially lost because of the low dynamic range when imaging. We find that almost all the research efforts try to avoid the “troublemaker” (the sun) rather than directly tackling the problem to improve the dynamic range.

In addition to the continuous improvement of the acquisition device of an all-sky image, an enormous amount of research effort has been focused on optimizing the cloud detection algorithm [9,11,12]. Most cloud detection algorithms focus on the scattering difference between red and blue wavebands caused by cloud particles and atmospheric molecules [13,14,15]. The clouds and the sky background are discriminated through the ratio of the two channels.

However, this method is less reliable and flexible since the ratio is set artificially and may not work under complex meteorological conditions (fog, haze, etc.). The authors of [16] reported the adjustable red green difference (ARGD) feature using red and green channels to distinguish sunlight interference in the image. Kruakaew et al., proposed an algorithm with brightness reduction to deal with the influence of the glare in the circumsolar region [17]. Tao fa et al. [10] also explored this field, applying the optimized U-Net model of the convolutional neural network (CNN) in the cloud detection algorithm to reach a convergence through the large sample training process, which acquires a higher operating frequency and a more stable observation effect. In general, previous research on cloud detection has focused on the scattering wavelength or intensity information of all-sky imaging, which is easily affected by complex meteorological conditions. In fact, the information extracted from all-sky imaging includes much more than wavelength and intensity. In fact, the cloud cover assessment does not make the best of sky polarization, mainly because of the difficulty in cloud layer extraction with a single piece of polarization information. Although Horvath et al., made some efforts in sky polarization information at an earlier stage [18] and Kreuter et al., presented a commercial digital camera with a fish-eye lens and a rotating polarizer [19], their experimental results were not satisfactory due to the limitations of immature polarization imaging equipment. Eshelman et al., verified that the ground-based all-sky polarimeter system reliably determines the cloud thermodynamic phase [20] but the system is unavailable for cloud detection.

In this paper, we propose a cloud detection system and a method based on all-sky polarization imaging. Inspired by the previous research work on polarization navigation, we use the polarization camera to procure all-sky images. Different from the reported research directly using polarization information, we defined the normalized polarization degree difference index (*NPDDI*) to achieve cloud selection through the difference in the degree of polarization (*DoPs*) between cloudy sky and clear sky. Due to the characteristics of polarization dimension, the stability of cloud detection can be improved to some extent, and highly accurate results can be obtained even under complex meteorological conditions. Furthermore, a fisheye lens is used for wide-angle imaging of the whole sky and the HDR all-sky imaging is probed by fusing two images (underexposed and overexposed). In this way, incomplete detection and the complexity caused by the sun blocking devices can be fundamentally avoided and the adverse effect on the accuracy of cloud detection can be eliminated to some extent. Moreover, clouds with different optical thicknesses can be extracted with different *NPDDIs* in this paper, which is not possible with previous cloud detection methods.

## 2. Theory and Method

### 2.1. Sky Polarized Light Distribution Pattern

In 1809, Arago discovered the polarization phenomenon in the sky. Sunlight is scattered by gas molecules in the atmosphere before reaching the Earth’s surface, and the scattered light is partially polarized, which can be explained by the Rayleigh scattering theory.

**Figure 1:**
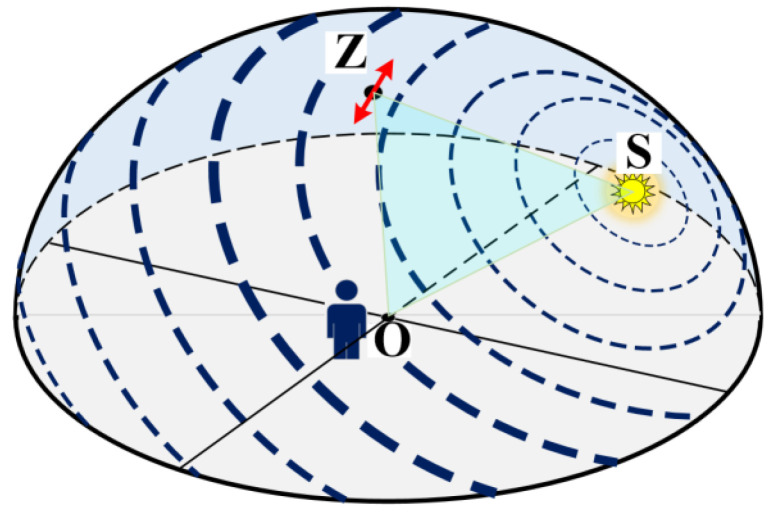
The sketch map of the skylight polarization pattern; the thickness and orientation of the arcs indicate the degree and direction of polarization, respectively.

According to the Rayleigh scattering theory, the polarization pattern at any point on the celestial sphere is associated with the sun’s position. Eshelman et al., compared the same set of all-sky images of Stokes parameters and the derived *DoP* and angle of polarization (*AoP*) in different planes of reference [21]. The polarization pattern of the full-view skylight is depicted in Figure 1, where O is the observer, Z is the zenith of the celestial sphere, and S stands for the sun. The dashed circle lines indicate the distribution of the polarized direction, which is perpendicular to the plane determined by the sun (the point S), the observer (the point O), and the point observed on the celestial sphere (e.g., point Z in Figure 1). Its orientation indicates the direction of the polarization distribution, and the thickness indicates the relative intensity of the *DoP* at different positions in the sky. As suggested by the results in Figure 1, the value of *DoP* reaches a maximum when the angle between OS and OZ is 90 degrees.

### 2.2. Real-time Measurement of the Sky Polarization Pattern

To obtain the polarization distribution pattern of the skylight, at least three images of the sky in different polarization directions are required, and real-time measurement requires that the three images be obtained at the same time. In 2015, Wenjing et al., realized the simultaneous acquisition of images in the three polarization directions with only one snapshot based on the structure of the light field camera [22]. However, the system cost of this method is relatively high, which limits its further application.

In 2017, Sony Corporation of Japan successfully realized the stable mass production of the high-performance polarization imaging chip (IMX250-MZR). The surface of each pixel in the chip is covered with a polarizing film. The directions of the four polarization films corresponding to the adjacent four pixels are different at 0°, 45°, 90°, and 135°. These four pixels constitute a “macro pixel” to measure the polarization information of one point, and the combination of the gray values can be used to obtain the Stokes vector $\vec{S}(I,Q,U,V)$, and then solve the polarization degree and polarization angle information of the corresponding observation point [22]. The calculation method is shown in the following equations:

(1) $$\left(\begin{array}{l} I\\ Q\\ U\\ V \end{array}\right)=\left(\begin{array}{c} \left(I_{0^{\circ }}+I_{45^{\circ }}+I_{90^{\circ }}+I_{135^{\circ }}\right)/2\\ I_{0^{\circ }}-I_{90^{\circ }}\\ I_{45^{\circ }}-I_{135^{\circ }}\\ 0 \end{array}\right),$$

(2) $$DoP=\frac{\sqrt{Q^{2}+U^{2}+V^{2}}}{I}=\frac{\sqrt{Q^{2}+U^{2}}}{I},$$

(3) $$AoP=\frac{1}{2}\arctan(\frac{U}{Q}),$$

In this article, we use a camera with an IMX250-MZR polarization imaging chip to acquire the sky polarization image.

### 2.3. The Principle of Cloud Detection with the Sky Polarization Pattern

When the sky is clear, skylight is polarized, mainly caused by Rayleigh scattering, which occurs in gas molecules located in the near-surface troposphere and the multiple scattering of light by clouds, aerosols, and the surface. Pust and Shaw et al., used a dual-field imaging polarimeter to study the influence of the aerosols and subvisual clouds on the degree of linear polarization [23]. However, the main component of clouds is water vapor, not gas molecules, and the direction of linear polarization also differs by the phase of clouds (comprised of liquid droplets or ice particles) [8]. The Stokes vector of transmitted light $\vec{S_{t}}(I^{\prime },Q^{\prime },U^{\prime },V^{\prime })$ under arbitrary illumination of a cloudy layer can be described using the following formulas [24]:

(4) $$\vec{S_{t}}(\mu ,\psi -\psi_{0})=\hat{T}(\mu ,\mu_{0},\psi -\psi_{0})\vec{F},$$

(5) $$\vec{S_{0}}(\mu ,\psi -\psi_{0})=\frac{1}{\mu_{0}}\delta (\mu -\mu_{0})\delta (\psi -\psi_{0})\pi \vec{F},$$

where μ is the cosine of the viewing angle, $\mu_{0}$ is the cosine of the solar angle, ψ is the azimuth of the sun, and $\psi_{0}$ is the azimuth of the observer. The Stokes vector of the incident light and the transmission matrices of cloudy media are represented by $\vec{S_{0}}(\mu ,\psi -\psi_{0})$ and $\hat{T}(\mu ,\mu_{0},\psi -\psi_{0})$, respectively. The first element of vector $\vec{F}$ stands for the net flux of solar beam per unit area of a cloud layer. In particular, the transmission matrices depend on the optical thickness and microstructure of the cloud layer [24]. Therefore, after being reflected by clouds, sunlight no longer has polarized properties, so we can separate the cloud from the sky background. Furthermore, the thicker the clouds, the stronger their ability to block sky background light radiation and the greater the attenuation of the optical radiation *DoP*.

**Figure 2:**
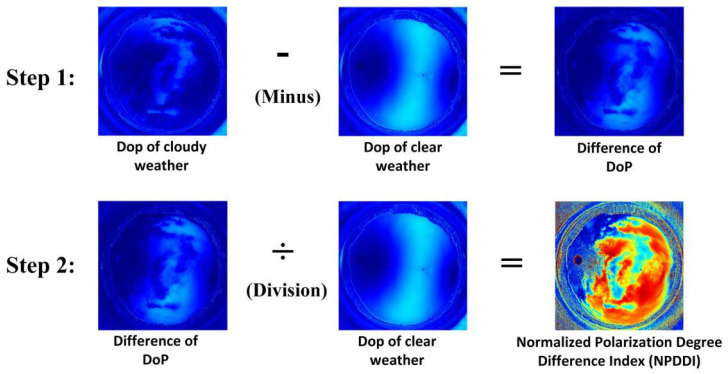
Flow chart of the cloud volume detection algorithm based on skylight *DoP* distribution. The final output of the algorithm is the *NPDDI* distribution result. The value range of the result is [0, 1], and the larger the value, the thicker the clouds.

However, as mentioned above, the *DoP* of the entire sky exhibits a non-uniform distribution: in some areas of the sky, the *DoP* can be 50% or more, while in other areas, the *DoP* may be only 5% or even lower. Therefore, just from the absolute *DoP* distribution of the sky, it is not easy to obtain accurate results of cloud distribution. We need to normalize the results of the polarization reduction caused by clouds. The entire process flow can be divided into two main steps, as shown in Figure 2. Step 1 is a differential process, which suppresses non-uniform distribution of the *DoP* to a large extent. Step 2 adopts the normalization method to highlight the difference in the *DoPs*, so we can extract the cloud layers more accurately and efficiently.

The algorithm flow in Figure 2 can be described with the following formula:

(6) $$NPDDI=Abs(DoP_{\_cloudy}-DoP_{\_clear})/DoP_{\_clear},$$

where *Abs* means absolute value, *DoP_cloudy* is the result of the all-sky *DoP* under cloudy weather conditions, *DoP_clear* is the result of the all-sky *DoP* of clear sky at the same solar elevation and azimuth conditions, and *NPDDI* is the normalized polarization degree difference index. Meanwhile, to reduce the influence of image noises on the accuracy of *NPDDI* calculation, several image processing methods are applied, such as the median filter and the average method. As suggested by the results, the thicker cloud layer has a larger *NPDDI*. Therefore, the algorithm can be used not only to detect the existence of clouds, but also to distinguish their different thicknesses. Through this algorithm, we can extract clouds with different optical thicknesses by adjusting the *NPDDI* value.

Although clouds play a dominant role in the attenuation of polarization of light radiation, other factors, such as aerosols and surface reflectance, also affect the all-sky *DoP*. When studying the effect of surface reflection on the all-sky *DoP*, Pust and Shaw pointed out that a reduction in the maximum *DoP* of the atmosphere is closely related to the multiple scattering of aerosols and the multiple reflections of the ground, which leads to the overall depolarization. Therefore, in this paper, to ensure the reliability of the cloud detection method, we carry out detection during periods when there are similar levels of atmospheric aerosol [23,25].

### 2.4. Eliminate the Effects of Strong Solar Interference

The existing high dynamic imaging technology is mainly divided into real-time and non-real-time directions. Because the polarization camera used in this experiment has a high acquisition frame rate (71 fps) and the sky scene and the solar elevation angle change slowly with time, the difference between the two images taken sequentially is almost negligible. Thus, high dynamic imaging can be composited with two images taken continuously but with different exposure times (one overexposure and one underexposure). The concrete processing procedure involves extracting moderately exposed portions of the two images and merging them into a complete high dynamic picture.

The principle of image fusion is as follows: For the two macro pixels (consisting of four adjacent pixels) corresponding to the same position in two images, calculate the average gray level of each macro pixel and select the average value closer to the moderate one, which is called the median value of the gray image. Since the exposure amount of this macro pixel is more appropriate, the polarization result calculated by this macro pixel is more accurate. The median value of the gray image is related to the depth of the image bit we selected. If the image bit depth is 8 bits, the median value of the gray value is 128, and if the image bit depth is 10 bits, then the value is 512.

## 3. Experiments and Results

**Figure 3:**
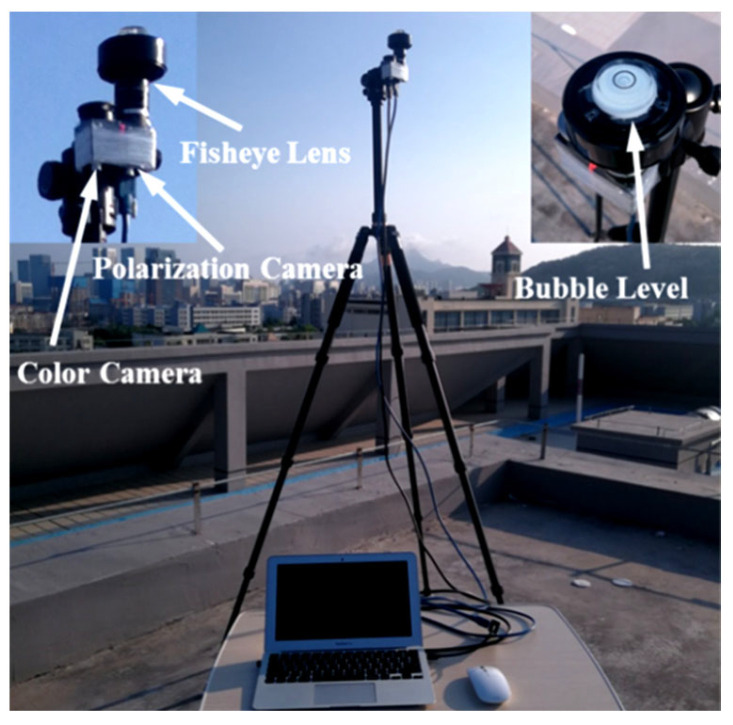
The experimental setup of the system installation. In addition to contrasting the cloud detection accuracy, the color camera provides a good basis for correcting the sun’s azimuth, since the feature points will be more obvious in the color image.

In the experiment, we used a camera based on Sony’s IMX250-MZR polarizing chip to acquire the sky’s polarization pattern in real time. At full resolution (2448 × 2048 pixels), the maximum frame rate is 71 Hz. To obtain the polarization information of the entire sky, we used the fisheye lens (brand: Fujinon; model: FE185C057HA-1; focal length: 1.8 mm) with a 180° view. Another color camera with the same lens was used to simultaneously acquire the color image of the sky to compare the accuracy of the cloud detection results. Figure 3 displays the experimental setup. The bubble level in the figure is used to adjust the camera’s shooting direction to ensure that the camera is always toward the right above.

**Figure 4:**
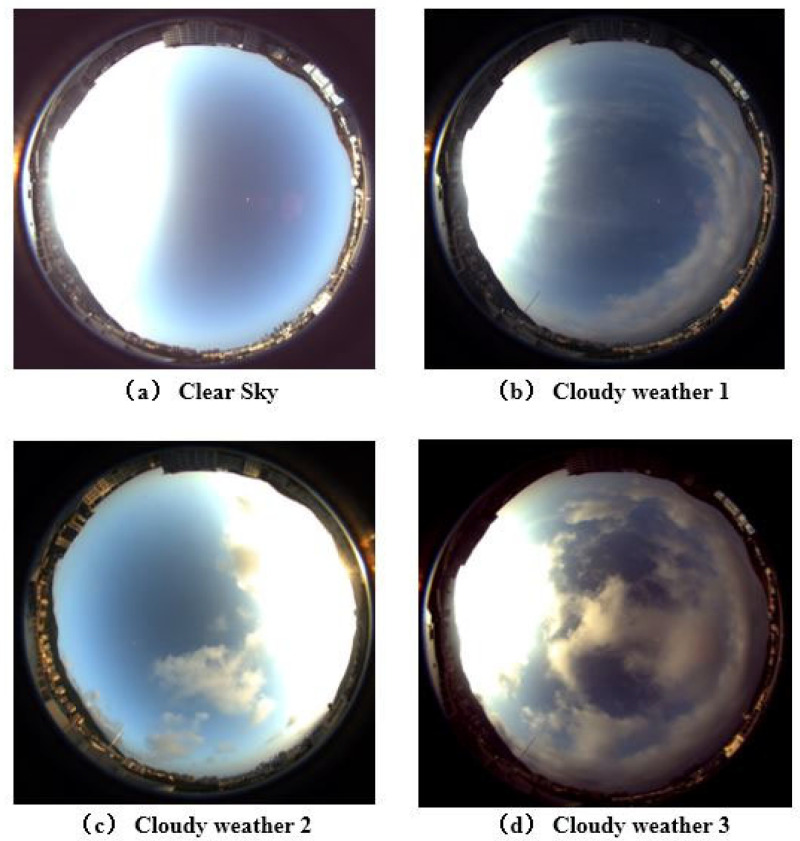
Real shot of the sky under different cloud conditions. When the sun is clearly visible in the sky, about one-third of the cloud information in the image will be lost (considering the distortion of the fisheye lens, this proportion will only be greater in reality). To solve this problem, traditional methods need to rely on sun-blocking devices, while we use HDR image fusion to incomplete the missing image details.

To test the accuracy and performance of our sky cloud detection method under different weather conditions, we carried out a series of experiments under clear and cloudy sky. These experiments were divided into different groups according to different weather conditions. Although the all-sky *DoP* can be more accurately determined by quantitative measurements and calculations, such as aerosol optical depth information, no further quantitative analysis was made, mainly due to the limitations of experimental conditions, and we expect to present a more detailed analysis in the future. Figure 4 shows four real shots of all the weather conditions.

### 3.1. Establishing a Polarization Distribution Database in Clear Sky

To ensure the preciseness of the cloud detection method, it is necessary to obtain the polarization pattern of the clear sky when the sun is at different elevation angles, the aim being to establish a comparison database, which means that the database images store all elevation angles of the sky polarization. Furthermore, the effect of the change in solar azimuth can be easily eliminated since different solar azimuths will only cause horizontal rotation of the sky polarization image under the same solar elevation angle, and the overall polarization distribution pattern remains unchanged.

The experimental site is on the roof of the Teaching Building, and the precise location is 120.488479° east longitude and 36.124712° north latitude. The experiment collects the full sky image almost at the same time and place every day. Due to the influence of the Earth’s rotation and revolution, the solar altitude and azimuth will change slightly at the same time every day. During the days of experiments, to eliminate the error caused by the sun’s position, we use the astronomical software to calculate the solar elevation angle every day. The exact acquisition time is adjusted slightly according to the change in the solar altitude to ensure that the images are captured at the same solar altitude. Meanwhile, even if the sun’s elevation angle is identical, the solar azimuth may be different. Fortunately, the influence of the solar azimuth in these images can be easily eliminated by image rotation.

**Figure 5:**
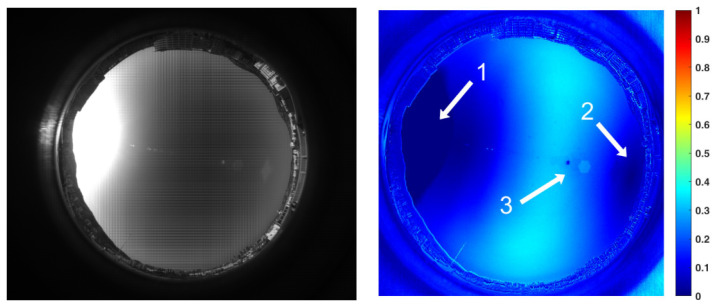
Exposed sky image obtained by the polarization camera and the corresponding polarization degree distribution results.

Figure 5 shows a frame of the sky image acquired by the polarization camera in our experimental system and the corresponding *DoP* distribution image. From this image, it can be seen that the area near the sun was severely overexposed, resulting in a zero value of the *DoP* region (i.e., the area indicated by arrow 1 in Figure 5). The degree of polarization of the region indicated by arrow 2 is also close to zero, consistent with the prediction of Rayleigh scattering theory. Moreover, it should be pointed out that the bright spot indicated by arrow 3 in Figure 5 is glare or ghost caused by strong sunlight entering the lens.

**Figure 6:**
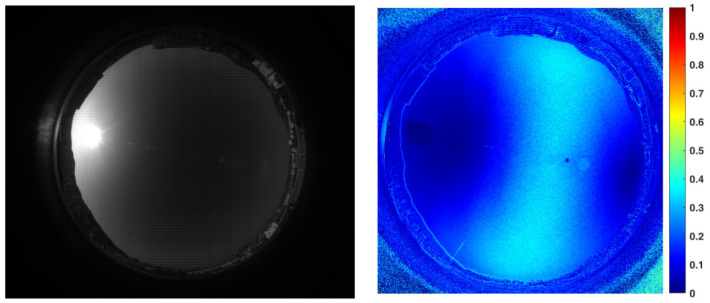
Underexposed sky image obtained by the polarization camera and the corresponding polarization distribution results.

To decrease the overexposed area in the image, we reduce the exposure time in the second capture, and Figure 6 displays the related *DoP* distribution map obtained. As can be seen from the figure, since most of the areas in the image are underexposed, the *DoP* distribution result has greater noise compared to Figure 5, which is disadvantageous for the later work of high-precision cloud detection.

**Figure 7:**
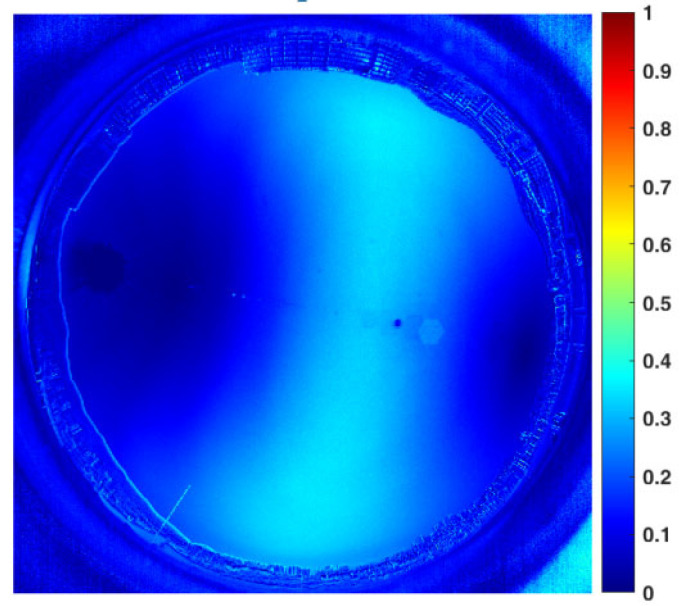
Sky polarization distribution results obtained by the image fusion algorithm. To compensate the mismatch of the cloud distribution caused by the time difference between the overexposed and underexposed images, we use an external trigger for timing control, and the time difference is compressed to less than 200 ms. Thus, the mismatch can be ignored.

We use the HDR image fusion method based on double exposures to select the best group of macro pixels that obtain the optimum exposure from both the overexposed and underexposed sky images. The complete *DoP* distribution result can be obtained in the full field of view, as shown in Figure 7. Not only is the overexposed area significantly reduced, but the image noise is obviously suppressed too. By sacrificing shooting efficiency, a non-real-time image sequence with artificially controllable exposure is obtained, and a high calibration accuracy can be guaranteed according to the accurate correspondence between the exposure time and the image intensity. With the method mentioned above, the *DoP* images are recorded with the sun at different elevation angles in sunny weather, so the comparison database for cloud detection can be established.

### 3.2. Cloud Detection Results under Partly Cloudy Conditions

This experiment was carried out at 06:45:12 on 12 June 2019, and the weather was partly cloudy. The corresponding solar elevation and azimuth angles can be acquired using the astronomical software and are 22.4547° and −77.0367°, respectively. Then we can find the *DoP* image of a clear sky with the same solar elevation angle as a comparison template from these data.

**Figure 8:**
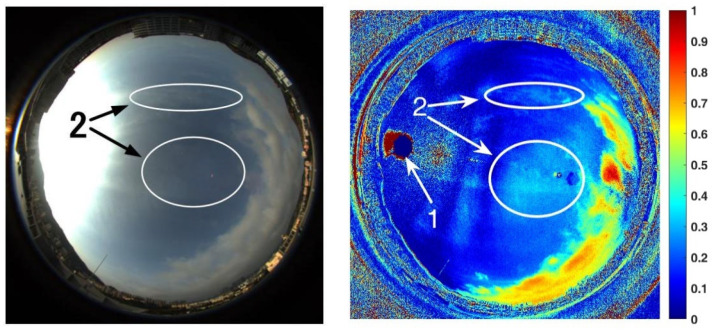
Color image of the sky cloud distribution and the corresponding *NPDDI* distribution results. The optical thickness of clouds can be distinguished by different *NPDDI* values, reflected in the *NPDDI* distribution image as the corresponding color in the color bar.

Figure 8 shows a frame of the sky image acquired by a color camera and its corresponding *NPDDI* distribution image. The different colors in the figure represent different *NPDDI* values. The areas with higher *NPDDI* values indicate the existence of cloud, and the larger the *NPDDI* value, the thicker the cloud layer. The area indicated by arrow 1 is also overexposed by sunlight, and the area with extremely thin clouds in the sky is indicated by arrow 2. The proposed cloud detection algorithm based on the *NPDDI* method can not only distinguish the thicker clouds from the sky background but also identify the extremely thin clouds. This is a good demonstration of our algorithm’s high sensitivity in cloud detection.

**Figure 9:**
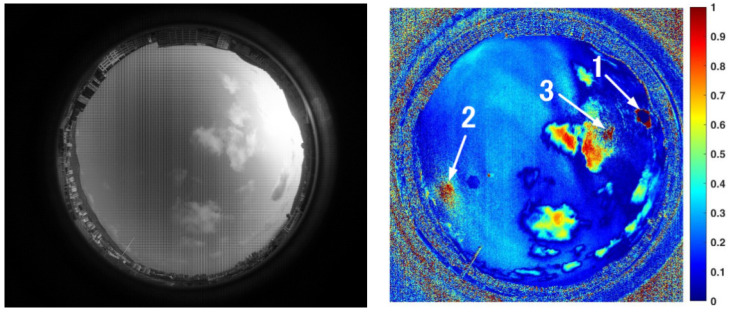
Polarization image under a partly cloudy condition and the corresponding *NPDDI* distribution result. The lack of polarization information brought by the Arago point and the Babinet point can be more clearly displayed.

The second set of experiments took place at 17:55:35 on 11 June 2019, when there was a small amount of clouds in the sky. At that moment, the solar elevation and azimuth angles were 13.4926° and 71.1388°, respectively. From these data, we procured the comparison template polarization image of a clear sky with the same solar elevation angle. Figure 9 shows one of the sky images acquired by a polarization camera and its corresponding *NPDDI* distribution image. Arrow 1 in the figure indicates the overexposed areas, while arrows 2 and 3 indicate the Arago point and the Babinet point of the sky [26,27,28]. At these two points, the polarization of the sky is almost zero, because of which, the cloud detection results represented by the *NPDDI* are relatively low in accuracy. Owing to the lack of polarization information near the neutral points, cloud detection methods using polarization information may face a similar problem [23]. A comparison of the two figures in Figure 9 shows a good correspondence between the cloud layers and *NPDDI* contribution, and the high cloud detection accuracy of the entire sky is demonstrated.

### 3.3. Cloud Detection Results under Cloudy Conditions

The experimental time of this group was 07:00:04 on 12 June 2019, and there was a large amount of clouds in the sky. At that moment, the solar elevation and azimuth angles were 25.3992° and −78.9566°, respectively. These data make it possible to find a polarization image of a clear sky with the same solar elevation angle as a comparison template.

**Figure 10:**
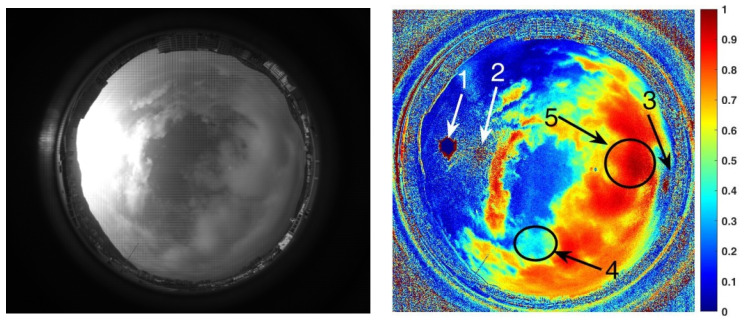
Cloudy sky image acquired by the polarization camera and the corresponding *NPDDI* distribution results. The cloud segmentation effect is obvious, and so is the comparison between thin clouds and thick clouds.

Figure 10 shows one of the sky images acquired by a polarization camera and its *NPDDI* distribution image in this group of experiments. Arrow 1 indicates the position of the sun. Arrows 2 and 3 indicate the Arago point and the Babinet point of the sky, respectively, and arrows 4 and 5 indicate the areas of the thin and thick clouds, respectively. It can be seen clearly that the cloud detection algorithm we proposed can not only distinguish the cloud from the sky background but also provide some quantitative optical thickness information on the cloud layer. If we set a threshold in the *NPDDI* image, a cloud layer thicker than a specific value can be selected from the image.

**Figure 11:**
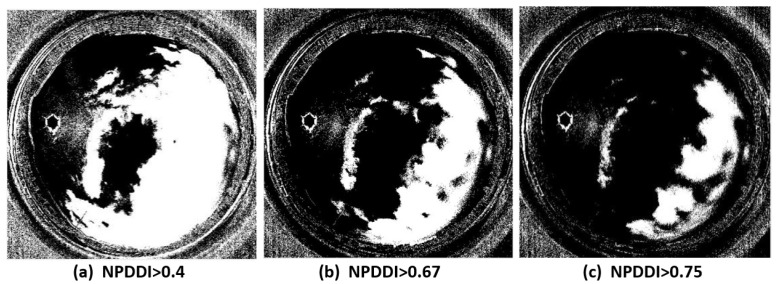
Cloud segmentation results when the *NPDDI* is larger than 0.4, 0.67, and 0.75, respectively. To show the segmentation effect of different *NPDDI* values more clearly, the results are transformed into binary images.

For images in Figure 10, we choose different thresholds of *NPDDI* to extract clouds thicker than different levels. Figure 11a–c show the cloud screening results, corresponding to the cloud segmentation results when *NPDDI* is >0.4, >0.67, and >0.75, respectively. The larger the *NPDDI* value set, the thicker the cloud layer selected, and the smaller the recognition area. The traditional cloud detection methods [13,14,15] use color information to discriminate the cloud layer. Specifically, as long as there are clouds in the sky, the color is certainly different from the sky background, regardless of the cloud’s thickness. Thus, all the areas will be selected irrespective of the optical thickness of the cloud, and the recognition result with the traditional methods is similar to Figure 10. Different from the traditional methods, with the ability of the *NPDDI*-based cloud detection method to distinguish the cloud optical thickness, we can eliminate the interference by the thin cloud layer and select the truly meaningful cloud layer area. Therefore, the accuracy of the cloud detection result can be significantly improved, which is of great significance for weather forecasting.

## 4. Discussion

### 4.1. Exploring Work on Cloud Detection Using Sky Polarization Angle Distribution

**Figure 12:**
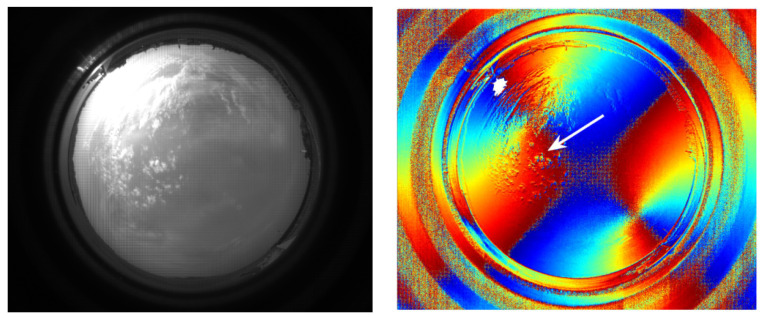
Cloudy sky image acquired by a polarization camera and the corresponding *AoP* distribution results. The presence of clouds has little effect on the distribution of the original *AoP* in the sky.

Pust and Shaw pointed out that the deviations of the cloud *AoP* from the clear-sky pattern depend on the phase of the cloud (composed of liquid or ice crystals), the relative brightness of the scattered light of the cloud, and the blocking of the sun by clouds [23]. In the experiment, we also tried to use the sky polarization angle distribution information to extract the cloud layer from the sky background, but we have not found a suitable method, the main reason being that the interference of the cloud layer in the sky polarization angle distribution has not yet been found to follow a clear law, compared with the sky polarization degree. Figure 12 shows a frame of the all-sky images acquired by the polarization camera and the corresponding polarization angle distribution information. The existence of the cloud layer causes an error in the distribution of the sky polarization angle (as indicated by the white arrow), but this error is not directly related to the optical thickness of the cloud layer.

**Figure 13:**
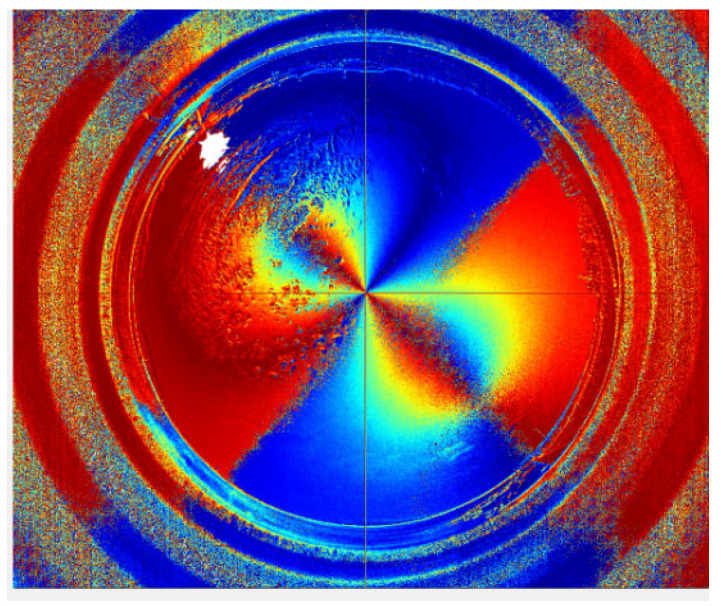
The redrawing result of the *AoP* distribution according to the new coordinate system.

We have redrawn the new coordinate system according to Figure 12 [29], and the result is displayed in Figure 13. It indicates that the distribution of the polarization angle of the sky has a certain degree of robustness, and the symmetry axis of the pattern clearly points in the direction in which the sun is located. Furthermore, the existence of the cloud layer does not substantially affect the distribution law of the polarization angle as a whole, which is advantageous for achieving polarized light navigation and disadvantageous if used to achieve cloud volume detection.

### 4.2. Other Problems in the Experiment

**Figure 14:**
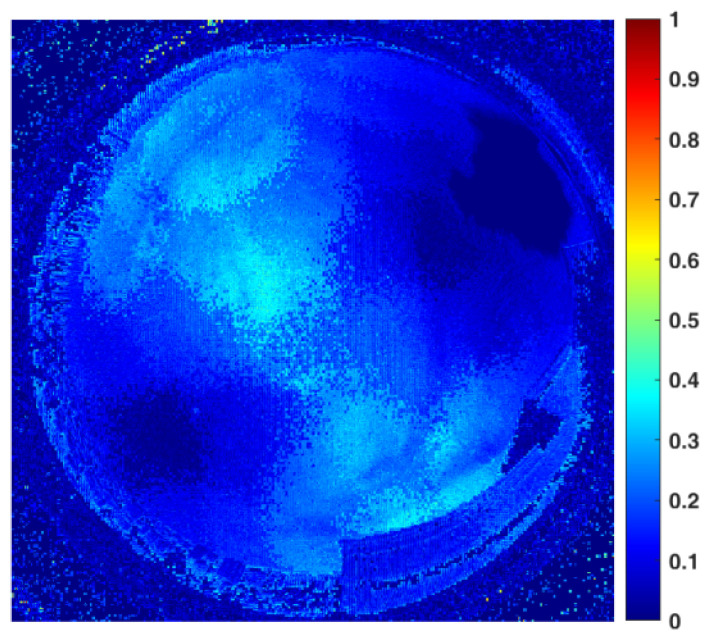
The results of the polarization distribution of a clear sky. The original image acquired by the polarization camera is saved in .jpeg format. Due to image compression, there is a significant error in the distribution result of the sky polarization degree.

In this experiment, to acquire the images of a polarized sky, we used a polarization imaging chip. We came to the following conclusions:

1. Considering that the polarization distribution of the sky is a slowly changing process, when we save the image acquired by the polarization camera in .jpeg format, the polarization measurement has a large error with a significant “layering” phenomenon. As shown in Figure 14, since the .jpeg image is compressed, the polarization profile under a clear sky has a certain loss. Therefore, in implementing cloud detection using the polarization camera, it is necessary to save the image in .bmp format, which can ensure accuracy to the greatest extent.
2. If the lens is not in precise focus, the polarization measurement of the sky will also have a large error. Since we use the polarization camera in our experiments to calculate the polarization information through four adjacent pixels, the target light received by the adjacent pixels is aliased if the lens is out of focus. Thus, there will be an error in the gray value of each pixel, which will affect the accuracy of the final polarization measurement result.
3. Since the above experiments are all carried out when the sun is at a lower elevation angle, the strong polarization effect of the sky enables our system to provide better cloud detection performance. However, when the sun is at a higher elevation angle, the sky polarization effect is weakened correspondingly, which reduces not only the difference between the polarization characteristics of the cloud layer and the sky background, but also the accuracy, accordingly.

## 5. Conclusions

The experimental results show that the proposed method has the following advantages compared with the traditional methods:

1. The cloud measurement of this system has high real-time performance. The cloud distribution data change rapidly as the clouds in the sky move and change. Since the calculation amount involved in our method is small, the related calculation procedure can be neglected. Thus, the system will achieve a high frequency of cloud detection, and it can fully adapt to the rapidly changing weather conditions.
2. The cloud measurement results of this system are less affected by overexposure to the sun. Due to the adoption of multi-frame different-exposure image fusion methods, the dynamic range of sky imaging is improved, significantly reducing the influence of solar glare on the accuracy of polarization measurement results.
3. The method can not only identify the cloud but also distinguish clouds of different optical thicknesses, while the existing cloud detection methods based on color recognition have certain limitations in distinguishing the optical thickness of cloud layers. Furthermore, the traditional methods also include thinner cloud layers in the cloud identification range, which obviously increases the error in cloud volume detection.

The existing problems and the improvements that can be made are as follows:

1. The cloud detection accuracy is unsatisfactory in the areas near the Babinet point and the Arago point.

In the vicinity of the Babinet point and the Arago point, the *DoP* is almost zero even in fine weather, which reduces the accuracy of cloud detection. Unfortunately, we have not yet found an effective solution to this problem.

2. The distribution information of the sky polarization angle was abandoned in the cloud detection method.

In fact, the image of the polarization angle distribution of the sky contains more information. In the next step, we will consider using the sky polarization angle distribution image together with the polarization degree distribution information to obtain more robust and accurate cloud detection results. For example, the sky polarization angle distribution has obvious symmetry, so we plan to obtain accurate results in terms of detecting cloud volume by evaluating the damage to the distribution symmetry.

## Data Availability

Data presented in this study are available on request from the corresponding author.
